# Case Report: Personalized management of HER2-positive breast cancer with advanced nodal disease during pregnancy: a clinical case and review

**DOI:** 10.3389/fonc.2025.1672751

**Published:** 2026-01-19

**Authors:** Carla Gullotta, Benjamin Walbaum, Elia Seguí, Marta López, Eduard Mension, Isaac Cebrecos, Meritxell Molla, Gabriela Oses, Xavier Bargalló, Sergi Ganau-Macias, Esther Sanfeliu, Fara Braso-Maristany, Montserrat Muñoz, Aleix Prat, María Vidal, Barbara Adamo

**Affiliations:** 1 Translational Genomics and Targeted Therapies in Solid Tumors, August Pi i Sunyer Biomedical Research Institute (IDIBAPS), Barcelona, Spain; 2 Cancer Institute and Blood Diseases, Hospital Clinic de Barcelona, Barcelona, Spain; 3 Department of Oncology, Fondazione Policlinico Universitario Campus Bio-Medico di Roma, Rome, Italy; 4 Department of Hematology Oncology, Pontificia Universidad Católica de Chile, Santiago, Chile; 5 Faculty of Medicine, Pontificia Universidad Católica de Chile, Santiago, Chile; 6 Facultat de Medicina i Ciencias de la Salut, Universitat de Barcelona (UB), Barcelona, Spain; 7 Department of Medical Oncology, Dana-Farber Cancer Institute, Boston, MA, United States; 8 Breast Oncology Program, Dana-Farber Cancer Institute, Boston, MA, United States; 9 Department of Obstetrics and Gynecology and Neonatology, Hospital Clinic of Barcelona, Barcelona, Spain; 10 Radiation Oncology Department, Hospital Clinic Barcelona, Barcelona, Spain; 11 Department of Radiology, Hospital Clinic, Barcelona, Spain; 12 Pathology Department, Hospital Clínic de Barcelona, Barcelona, Spain; 13 Molecular Biology CORE (CDB), Hospital Clínic de Barcelona, Barcelona, Spain; 14 Reveal Genomics, Barcelona, Spain; 15 Institute of Oncology (IOB), Hospital Quironsalud, Barcelona, Spain

**Keywords:** circulating tumor DNA, HER2DX, imaging during pregnancy, multidisciplinary approach, neoadjuvant therapy, pregnancy-associated breast cancer, targeted axillary dissection

## Abstract

**Background:**

Pregnancy-associated breast cancer (PrBC) poses complex challenges in diagnosis and treatment, particularly when associated with biologically aggressive subtypes and extensive nodal involvement. Management must be individualized, integrating oncologic urgency, fetal safety, and limited validated evidence in this unique setting.

**Case Summary:**

We present the case of a 36-year-old woman diagnosed during the second trimester of pregnancy with HER2-positive (HER2+), node-positive (cT2[m]N3a) breast cancer (BC). After a multidisciplinary team discussion, patient initiated anthracycline- and taxane-based neoadjuvant chemotherapy during gestation. Given its contraindication during pregnancy, anti-HER2 therapy was added postpartum, and surgery included nipple-sparing mastectomy with targeted axillary dissection (TAD) of clipped nodes. Pathology revealed minimal residual invasive disease in the breast and a complete axillary response, allowing omission of axillary lymph node dissection (ALND). Genomic profiling with HER2DX supported high-risk disease and informed systemic therapy with delayed anti HER2 therapy, and conservative axillary management (TAD without ALND) in cT2N3 PrBC, without compromising fetal outcome. The patient subsequently received adjuvant chest wall and nodal region radiotherapy plus trastuzumab-emtansine (T-DM1).

**Conclusion:**

This case underscores the value of personalized, multidisciplinary management in PrBC, particularly in patients with high-risk biologic features and advanced nodal disease. Integrating clinical judgment, genomic tools, and adaptive strategies, while accounting for gestational limitations, can optimize oncologic outcomes without compromising fetal safety.

## Introduction

Pregnancy-related breast cancer (PrBC) is the most common malignancy diagnosed during pregnancy (1). Although still relatively rare, its incidence has been steadily increasing, in line with rising breast cancer (BC) rates among young women and a trend toward delayed childbearing (2–4).

Histologically, biologically and more importantly, prognostically, PrBC does not appear to differ significantly from BC diagnosed outside of pregnancy, when adjusted by age, stage and subtype (5, 6). Though there still is a higher prevalence of triple-negative and HER2-positive (HER2+) subtypes (7). In recent years, treatment for both subtypes has evolved beyond chemotherapy to include immunotherapy and anti-HER2 therapy, respectively. However, these therapies are contraindicated during pregnancy, due to safety concerns (8–12). Thus, chemotherapy remains the only approved systemic treatment option during gestation. This highlights the need for new data to guide integration of emerging systemic strategies.

Predictive clinical biomarkers and genomic platforms support clinicians in tailoring treatment. To further personalize therapies, a growing array of new genomic platforms have emerged to better stratify risk and predict therapy benefit. Yet, there is limited data regarding their use in pregnant women.

Additionally, there is a growing interest in dynamic biomarkers during neoadjuvant strategies, to enable treatment (de)escalation, such as in-treatment imaging and circulating tumor DNA (ctDNA). However, imaging techniques face limitations during pregnancy, as irradiation is avoided and physiological breast changes, such as increased density and vascularity, hinder both clinical and radiological evaluations (13–15). Thus, ctDNA monitoring during and after neoadjuvant therapy, could be an alternative tool to aid in clinical evaluation and help identify tumor response to treatment (16).

Regarding the locoregional approach, significant advancements in axillary surgery have made it less morbid and more conservative, particularly in the context of high response rates after neoadjuvant therapy. Conservative surgical approaches, including breast-conserving surgery, sentinel lymph node biopsy and targeted axillary dissection (TAD), are feasible both during pregnancy and after delivery. However, adjuvant radiotherapy as part of the breast-conserving strategy should be postponed until after childbirth. Still, multidisciplinary care involving oncology, obstetrics, neonatology, radiology, and pathology remain critical to achieve optimal outcomes​.

We present a case that exemplifies these challenges and discuss their broader implications. We explored the use of HER2DX, a new genomic platform designed to support clinicians in tailoring treatment. Moreover, in this case we evaluated the importance of dynamic biomarkers during neoadjuvant strategies, such as in-treatment imaging, especially challenging in pregnant women, and the potential role of circulating tumor DNA (ctDNA). Finally, we adopted conservative surgical approaches, including targeted axillary dissection (TAD) after neoadjuvant therapy.

## Case presentation

**Figure 1:**
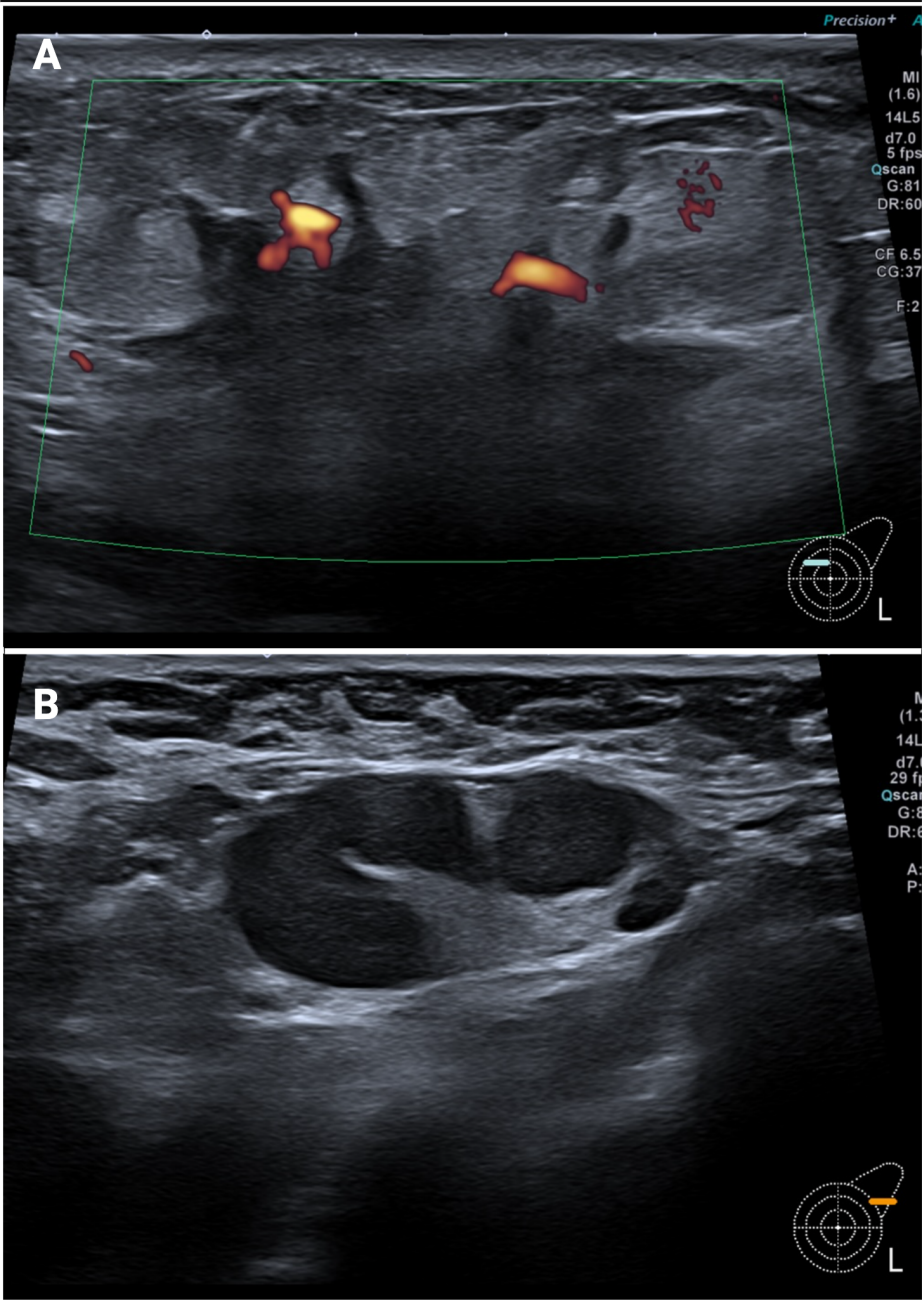
**(A)** Radiology assessments obtained at diagnosis. Breast ultrasound: spiculated mass highly vascularized (Doppler color positive). **(B)** Radiology assessments obtained at diagnosis. Breast ultrasound: enlarged axillary lymph node.

In April 2024, a 36-year-old pregnant woman at 22 weeks of gestation presented with progressive induration of the left breast accompanied by nipple discharge. She had no relevant comorbidities. Clinical breast examination and ultrasound, identified two tumor foci: a 45 mm nodule in the upper-inner quadrant and a 20 mm lesion in the upper-outer quadrant [**Figure 1A**], confirming a multicentric tumor: cT2(m). A biopsy of both foci confirmed the diagnosis of invasive carcinoma of no special type (ductal, NST), histologic grade 2 and with stromal tumor-infiltrating lymphocytes (TILs) of 2%; the immunohistochemical results were ER negative, PR negative, HER2+ (score 3+) and Ki67 proliferative index of 35%. In addition, an infraclavicular lymph node was cytologically positive, along with suspicious ipsilateral axillary lymph nodes, leading to a N3a nodal staging [**Figure 1B**]. Staging exams, including chest X-ray (with abdominal shielding) and abdominal ultrasound, ruled out distant metastases.

To further refine prognosis and guide therapeutic strategy, a HER2DX genomic test was performed. HER2DX provides three scores: the risk score estimates the probability of disease recurrence, the pCR score estimates the likelihood of achieving pathological complete response to neoadjuvant therapy, and the ERBB2 score measures the level of HER2 pathway activation, with each score helping to guide treatment decisions. (17) This genomic test revealed a high risk of recurrence (relapse risk score: 93), intermediate likelihood of pathological complete response (pCR) (pCR score: 65), and moderate *ERBB2* expression (*ERBB2* mRNA level score: 48). Based on these clinical and molecular data, patient was a candidate for multiagent neoadjuvant chemotherapy. With limited margin and scarce data for treatment de-escalation, given the advanced clinical stage, high molecular risk of relapse, young age, and potentially moderate response to anti-HER2 agents, considering its moderate HER2 “addiction”. Moreover, given the advanced nodal involvement and high-risk features, the case was promptly discussed in a multidisciplinary team (MDT) involving oncologists, breast surgeons, radiologists, radiation oncologists, obstetricians, and gynecologists. Together with the patient, the decision was made to proceed with neoadjuvant chemotherapy starting at 23 weeks of gestation.

Prior to starting therapy, and after a thorough discussion, the ultrasonographical visible lymph nodes lesions were clipped (one clip placed in the left supraclavicular level III node and three clips placed in level I axillary lymph nodes), as the patient insisted on opting to more conservative axillary surgery, despite the MDT recommendation for an axillary lymph node dissection (ALND). We discussed with the patient the potential oncologic risks associated with this decision; however, the MDT considered these risks acceptable in light of the evidence supporting TAD, the HER2DX test results suggesting a moderate likelihood of achieving a pCR, and the role of intermediate imaging assessments.

### Neoadjuvant chemotherapy during pregnancy

In April 2024, at 23 weeks of pregnancy, the patient began treatment with doxorubicin and cyclophosphamide, agents known to be safe in this setting, every 3 weeks for 4 cycles. A 3-week schedule was selected, despite her young age and risk, to ensure better tolerability and allow a prolonged anthracycline exposure during pregnancy, and thus postpone the second part of the neoadjuvant therapy with anti-HER2 agents, which must be avoided during pregnancy. This strategy maximized both treatment duration and gestational time. Steroid and antiemetic premedication were adjusted according to BC and pregnancy guidelines to minimize maternal and fetal side effects. The patient was weighed before each cycle to ensure accurate dosing. Normal fetal development was confirmed by ultrasound and fetal echocardiographic assessment before initiating treatment. Fetal growth, fetal development, and placental function were monitored biweekly through ultrasound and biometric parameters throughout treatment.

**Figure 2:**
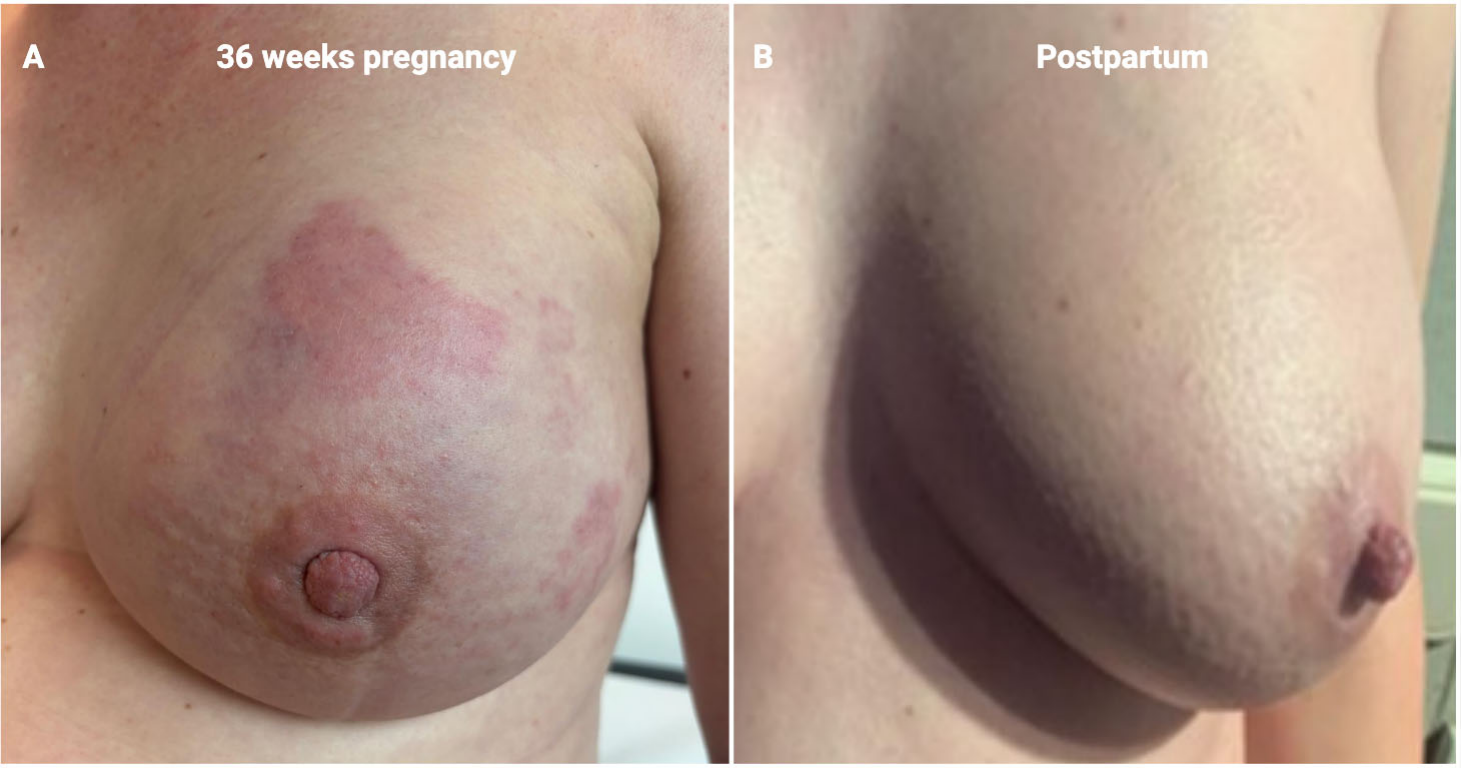
**(A)** Image of clinical response at 36 weeks of pregnancy, after 4 cycles of anthracyclines and 1 cycle of paclitaxel therapy. **(B)** Imagine of clinical response after completing neoadjuvant treatment.

After completing anthracyclines, in July 2024 she received her first dose of paclitaxel at week 36 of gestation. At this stage, clinical evaluation of the breast was challenging: the entire breast appeared increasingly inflamed, indurated, and swollen, with no clear signs of tumor shrinkage [**Figure 2A**]. Rather than remaining stable, the local condition seemed to worsen, raising concern for disease progression or treatment resistance. This presentation made clinical assessment unreliable, prompting the MDT to consider intensifying therapy by adding carboplatin after delivery, for the second phase of the neoadjuvant therapy, with paclitaxel and pertuzumab-trastuzumab.

At 37 weeks, in the first week of August she underwent induced vaginal delivery, which was uneventful and allowed for early chemotherapy resumption just 10 days later. The newborn was healthy, with a birth weight of 2645 grams (14th percentile) and normal neonatal examination, including appropriate Apgar scores. Single dose cabergoline was used to avoid breastfeeding. Following delivery, breast edema subsided within days. Only then did it become evident that the patient had achieved a profound clinical response, with marked reduction of palpable abnormalities in both the breast and axilla [**Figure 2B**].

### Continuing neoadjuvant chemotherapy and anti-HER2 therapy post-partum

In August 2024, one week after delivery, the patient resumed therapy with paclitaxel, now combined with trastuzumab and pertuzumab. Of note, these anti-HER2 agents had been excluded during pregnancy due to the known risk of oligohydramnios and fetal renal toxicity, particularly in the second and third trimesters. Despite limited sensitivity during systemic therapy a PET/CT scan was performed and confirmed the absence of distant disease. The patient also underwent genetic counselling and germline testing, which revealed no pathogenic germline variants in breast cancer-related genes.

**Figure 3:**
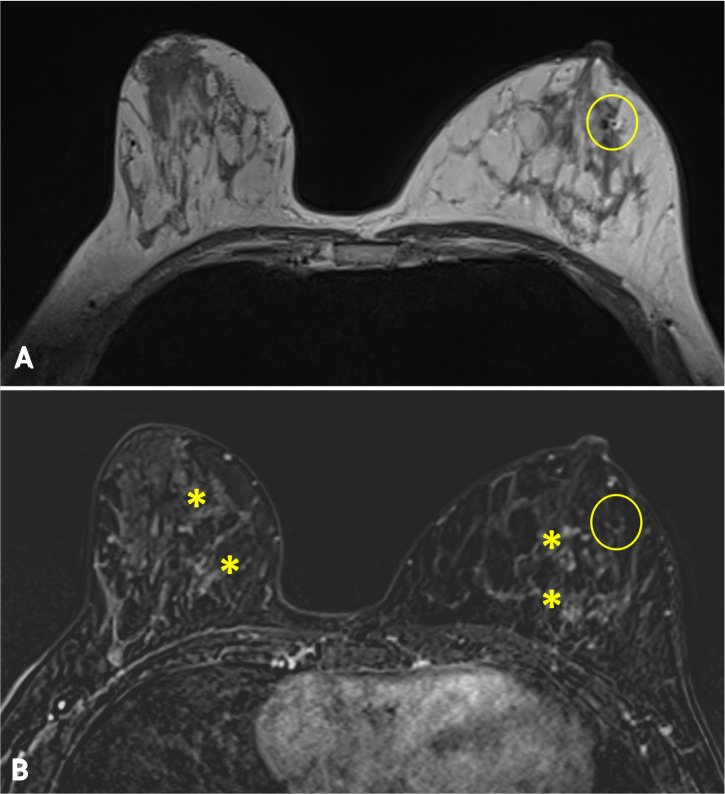
**(A)** Breast MRI after neoadjuvant chemotherapy. **(A)** Hydrophilic clip marker (circle) without **(B)** associated enhancement, compatible with complete response. Notice physiological parenchymal enhancement in both breasts (asterisks).

Finally, the clinical response was confirmed radiologically with a breast MRI after the end of neoadjuvant therapy, in October 2024, that described a radiological complete response [**Figure 3**].

### Breast surgery

In November 2024, after confirming negative germline mutation status and completing 12 weeks of paclitaxel, trastuzumab and pertuzumab, the patient underwent a nipple-skin-sparing left mastectomy with immediate expander reconstruction. Surgery included intraoperative assessment of sentinel lymph nodes using one-step nucleic acid amplification (OSNA) and TAD of the previously marked cN3 nodes. Pathology revealed a residual 3 mm invasive ductal carcinoma, grade 2, 10 mm of *in situ* carcinoma, and no residual axillary disease (0/4 lymph nodes). Final stage was ypT1a ypN0. After MDT discussion, in accordance with the patient’s preference, and given the axillary pCR, and the planned adjuvant radiotherapy, ALND was not performed.

**Figure 4:**
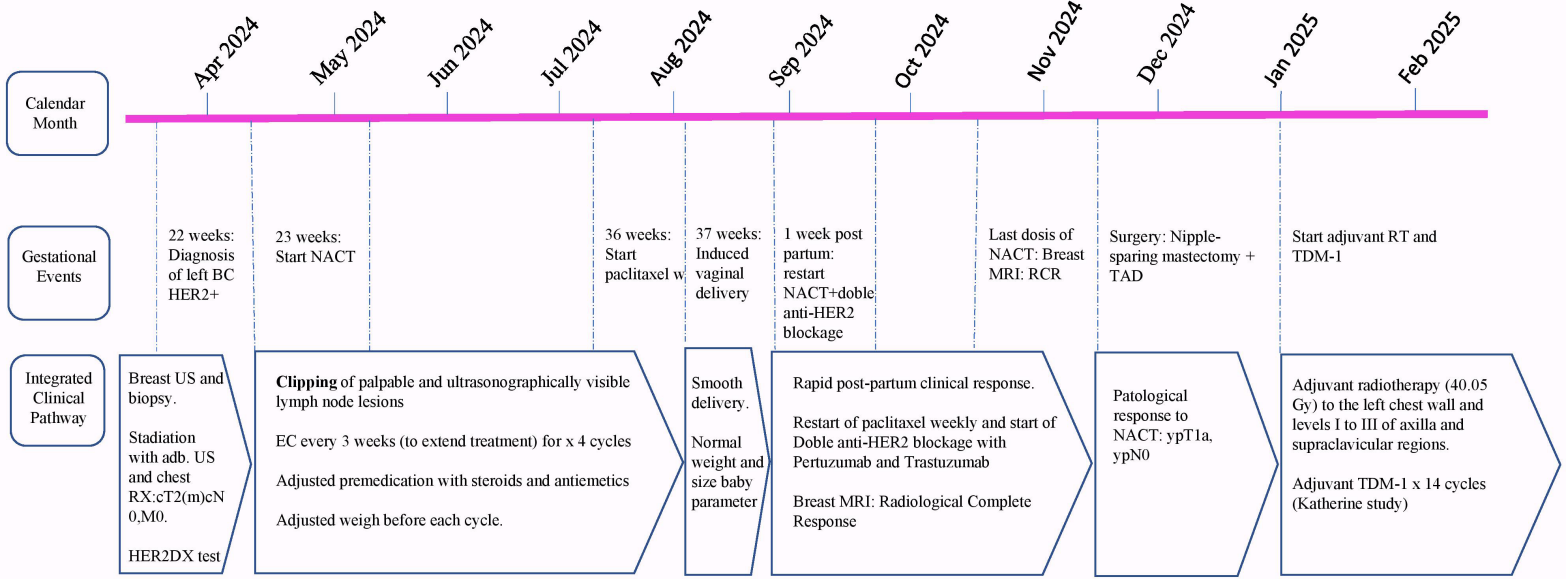
Clinical timeline of diagnosis and breast cancer treatment during pregnancy and after delivery.

In January 2025, she started adjuvant radiotherapy (40.05 Gy) to the left chest wall and levels I to III of axilla and supraclavicular regions. In fact, although recent results from the NSABP B-51 trial suggest of the safety of omitting regional nodal irradiation in patients achieving ypN0 after neoadjuvant therapy, in our patient, regional nodal irradiation was administered to compensate for the lack of surgical nodal clearance and ensure appropriate locoregional control. She is currently receiving adjuvant trastuzumab-emtansine (T-DM1) for 14 cycles, according to the KATHERINE trial protocol, which demonstrated that the use of T-DM1 in the adjuvant phase, in the presence of residual disease after neoadjuvant therapy, significantly improves event free survival (EFS) and overall survival (OS) compared with trastuzumab alone (18). The treatment timeline is summarized in **Figure 4**.

## Discussion

PrBC represents a unique clinical challenge, requiring a balance between optimal maternal oncologic care and fetal safety (19). In this case, the management of a patient with HER2+ disease posed further difficulties due to restriction in the use of standard of care systemic therapy during gestation. We will review the existing clinical guidelines, illustrate how they were applied in our case, and highlight the aspects in which our management diverged. This will allow us to underline current gaps that should be addressed in such complex clinical scenarios.

### Systemic treatment during pregnancy

In this case, the patient received a neoadjuvant regimen with anthracyclines and cyclophosphamide (AC) during pregnancy, as detailed below. In fact, chemotherapy regimens administered during the second and third trimesters are considered safe for the fetus, with no consistent evidence of long-term teratogenicity (20). Anthracycline-based regimens remain the cornerstone of treatment, and the addition of taxanes has been shown to be feasible and effective when initiated after the first trimester (20)​. According to the National Comprehensive Cancer Network (NCCN) guidelines, the combination of anthracyclines and taxanes is the recommended neoadjuvant regimen for HER2+ early BC during pregnancy (category 1A recommendation). It is important to note that in non-pregnant patients with HER2+ disease, anthracycline-sparing regimens have become the preferred option, with anthracycline-containing regimens now categorized as a 2B recommendation in the 2021 NCCN updates (21)​. This shift reflects growing concern over anthracycline-related cardiotoxicity in non-pregnant settings.

Moreover, limited data suggest that the use of platinum salts appears to be safe in this selected population. Several reports, primarily involving patients with ovarian cancer, show that carboplatin does not adversely affect short-term fetal outcomes. However, data on animal models warns of high transplacental passage, and clinical cases have reported an increased risk of ototoxicity in children exposed to cisplatin during gestation (22).

Anti-HER2 therapy was not administered during pregnancy, in accordance with current literature guidelines, as discussed in detail below. Targeted therapies such as trastuzumab and pertuzumab are contraindicated during pregnancy due to the risk of oligohydramnios, as fetal renal tissue expresses HER2. Their use is particularly risky beyond the first trimester, when fetal renal development and amniotic fluid production become critically dependent on HER2 signaling (12) However, monoclonal antibodies like trastuzumab are large molecules (>100 kDa) and require active Fc receptor–mediated transport, which is not functional until approximately 14 weeks of gestation. Consequently, accidental exposure during the first trimester is unlikely to result in significant fetal transfer (23).

Immune checkpoint inhibitors (ICIs) pose additional risks during pregnancy, notably through disruption of maternal-fetal immune tolerance. Extravillous trophoblasts (EVTs) (24), which mediate placental implantation, express high levels of programmed death-ligand 1 (PD-L1), contributing to maternal-fetal immune tolerance. The PD-1/PD-L1 axis plays a central role in allowing the maternal immune system to tolerate fetal alloantigens. PD-L1 negatively regulates maternal alloimmune responses, and its blockade could disrupt this tolerance, potentially leading to fetal rejection (25). Moreover, anti-PD-1 agents like pembrolizumab, now approved for neoadjuvant treatment in patients with TNBC, cross the placenta. Data from melanoma cases have reported significant fetal adverse events, including immune-related events and neonatal autoimmune disorders (26). A recent report by Baarslag et al. (27) described a 4-month-old infant who developed severe immune-related gastroenterocolitis following *in utero* exposure to pembrolizumab, which had been administered from the second trimester onward for maternal melanoma. The infant presented with intractable diarrhea and failure to thrive, requiring immunosuppressive therapy with corticosteroids and infliximab. Immunophenotyping revealed hyperactivated T cells with increased PD-1 and HLA-DR expression, consistent with ICIs-induced immune activation. This case underscores the potential for delayed-onset immune-related toxicities in neonates and highlights the serious risk of fetal immune dysregulation resulting from maternal ICIs therapy.

Given these emerging results, the use of these drugs during pregnancy should be avoided unless no alternatives exist, and maternal survival is at imminent risk. Multidisciplinary decision-making and close neonatal follow-up are essential if maternal treatment with ICIs is pursued during gestation.

Further research is needed to determine if delaying these newer and more effective targeted strategies may impact prognosis in pregnant patients, as historical data comparisons with non-pregnant patients did not include many of these novel options.

Alongside chemotherapy, supportive therapy is essential for the optimal management of pregnant. Although granulocyte colony stimulating growth factors (G-CSF) were not indicated in this case, they may be used during pregnancy when clinically justified. Transplacental passage of G-CSF has been documented, but available studies report no increase in fetal death or congenital malformations. Long-term follow-up of exposed children has shown no major neurological or cardiac abnormalities. Consequently, both short- and long-acting G-CSF formulations may be considered and used when clinically indicated, such as in the context of dose-dense chemotherapy regimens, as they do not appear to pose substantial fetal risk (28–31).

Antiemetic regimens must be adjusted during pregnancy. While ondansetron may be considered when necessary, it crosses the placental barrier. Current guidelines recommend avoiding its use, particularly in the first trimester, due to a small but significant increased risk of orofacial clefts and cardiac malformations​ (32). Premedication with antiemetic, like ondansetron, was carefully considered throughout the chemotherapy regimen, but in this case, it was ultimately not required. Furthermore, corticosteroid use for chemotherapy premedication also requires caution. Dexamethasone and betamethasone should be avoided, particularly before 10 weeks of gestation, due to their nearly complete placental transfer and association with teratogenic effects, mainly an increase of oral clefts (33)​. Additionally, prenatal exposure to dexamethasone has been linked to long-term neurobehavioral alterations (34). Methylprednisolone or prednisolone are preferred alternatives, as they undergo placental metabolism and result in lower fetal exposure (35). Accordingly, in this patient premedication with methylprednisolone was administered, given its established safety during pregnancy.

### Use of genomic tools

Emerging genomic tools, such as the HER2DX assay, may help to guide clinical decision-making in such complex scenarios. HER2DX combines clinicopathologic and transcriptomic features to generate prognostic (relapse risk) and predictive (pCR likelihood) scores (17, 36–40). In our case, the HER2DX test suggested a high risk of relapse with an intermediate probability of achieving a pCR and intermediate levels of *ERBB2*, prompting a recommendation for an intensified therapeutic approach. It should be noted that HER2DX, like other genomic prognostic tests, has not been validated in pregnant women. Therefore, while it provided additional information, its results should be interpreted with caution, as pregnancy-related changes in the tumor microenvironment could potentially lead to over-treatment.

Interestingly, genomic profiling studies suggest that BC diagnosed during pregnancy may display distinct molecular features. PrBC, in fact, more frequently exhibit basal-like and HER2-enriched subtypes, with increased expression of proliferation-related genes and downregulation of immune-related pathways, compared to age-matched non-pregnant counterparts (41, 42). This genomic landscape could contribute to the generally more aggressive behavior of PrBC. However, it is important to highlight that no study has specifically validated the prognostic performance of commercially available genomic signatures, such as Oncotype DX, MammaPrint, or HER2DX, in patients diagnosed during pregnancy​. Only one study evaluating GENE70 expression found no major differences between pregnant and non-pregnant cohorts (42). Furthermore, even in the broader population of young women, genomic arrays face certain limitations. In the pregnancy setting, ESMO experts suggest that genomic testing could be cautiously considered in pN0, ER-positive patients, to support de-escalation decisions. However, any decision based on genomic risk stratification must acknowledge the potential influence of pregnancy-induced microenvironmental changes, which could affect the assay’s predictive accuracy.

Additionally, while tumor-infiltrating lymphocytes (TILs) have traditionally been regarded as relevant prognostic biomarkers and predictive of increased response to treatment, their evaluation during pregnancy warrants caution (43). Studies suggest that TILs profiles in PrBC are altered and vary by gestational age, with increased regulatory T cell (Treg) populations early in gestation and dynamic changes as pregnancy progresses (43). High TILs levels during pregnancy may not necessarily indicate effective cytotoxic immune activity but could, in some cases, reflect a tolerogenic immune environment (43). In our case, this is consistent with the very low TILs level observed (2%), which may reflect the immune tolerance characteristic of pregnancy rather than a true lack of treatment responsiveness. In this context, genomic tools such as HER2DX that include immune gene expression with a strong B-cell-related immune signatures, could offer a more reliable measure of effective antitumor immunity (36). As B-cell and plasma cell signatures have been consistently associated with better prognosis and may better capture the quality of the immune response compared to TILs quantification alone (44).

While genomic assays offer promising avenues for risk stratification in PrBC, their use during pregnancy must be approached with caution, and results should be interpreted within a multidisciplinary framework that carefully integrates clinical, pathological, and gestational considerations.

It is also worth considering that the apparent aggressiveness of PrBC may not be solely due to inherent biological differences but could also be influenced by diagnostic challenges during pregnancy. Physiological changes, such as breast edema, can mask symptoms and potentially delay diagnosis. As a result, more aggressive tumors may be detected during pregnancy, whereas indolent tumors developing during gestation might only be diagnosed postpartum. This diagnostic bias underscores the need for further research to better understand biological changes occurring during pregnancy and their impact on BC.

### Response assessment and imaging

Another major challenge is clinical assessment of tumor response during treatment. In our case, during the third trimester, the breast appeared progressively more inflamed and edematous, rendering clinical evaluation unreliable. Pregnancy-related physiological changes, such as glandular hypertrophy and increased vascularity, can mask true tumor response (26). These changes also influenced the MDT decisions, leading to the consideration of intensifying the second phase of neoadjuvant therapy, by adding carboplatin, and eventually anticipating the timing of delivery. Only after delivery did clinical signs of response become evident, highlighting the importance of multimodal assessment and the potential role of imaging tools like MRI, which, although limited during pregnancy, can provide critical information postpartum. Considering the excellent post-partum MRI results, the MDT decided to proceed with the planned treatment regimen of paclitaxel, pertuzumab, and trastuzumab. Moreover, with the gaining traction of de-escalation strategies guided by mid-treatment imaging, as exemplified by evidence obtained in non-pregnant populations in the PHERGain and TRAIN-3 trials (45, 46), these and other approaches could be further evaluated in pregnant patients. Circulating tumor DNA (ctDNA) analysis is an emerging, minimally invasive biomarker that reflects tumor burden and response in real time in non-pregnant patients from the general population. Although its clinical use during pregnancy is still investigational, recent evidence confirms its biological feasibility. Turriff A et al, reported that among pregnant women with abnormal or unusual non-informative results from genome-wide noninvasive prenatal testing (NIPT), nearly half were found to have an underlying malignancy, including BC in 7.7% of cases. Among these patients, cell free DNA (cfDNA) showed distinctive patterns of copy number alterations, demonstrating that ctDNA may be detectable in maternal plasma during gestation (47). These findings underscore the potential of ctDNA analysis as a monitoring tool during pregnancy, although interpretation is complicated by the presence of fetal cfDNA and dynamic maternal-fetal interactions. If prospectively validated, ctDNA could complement clinical exam and ultrasound in assessing response or minimal residual disease during gestation, especially considering imaging limitations. Further research is needed to validate its clinical utility in PrBC (16).

### Surgery and axillary management and radiotherapy

The choice of breast cancer surgery during pregnancy should generally follow the same principles as for non-pregnant women, favoring breast-conserving therapy when clinically feasible (48). Immediate breast reconstruction with prosthetic implants can be considered, whereas autologous reconstruction is typically delayed until after delivery, considering pregnancy-related physiological changes (48). Importantly, the need for postpartum radiotherapy after breast-conserving surgery should not drive the decision toward mastectomy, as irradiation can safely be deferred until after delivery (48). Additionally, in women with plans for future breastfeeding, breast-conserving surgery may offer functional and psychological advantages over mastectomy (49). In our patient, however, mastectomy was performed due to the multicentric nature and large size of the tumor, making breast-conserving therapy impractical.

The decision to pursue a de-escalated axillary surgical approach using TAD was a critical aspect of this patient’s management. This strategy, combining sentinel lymph node biopsy with the removal of clipped nodes, is strongly supported and well established in the neoadjuvant setting for patients with limited nodal involvement at diagnosis. Although evidence in PrBC is limited and again largely focused on cN1 cases, our case underscores the importance of personalized surgical planning. This approach enabled a more conservative strategy when a complete nodal response was achieved (50). While prospective validation is still needed to confirm the safety of TAD in patients with extensive nodal disease (50), our MDT supported this individualized approach based on the patient’s strong preference to avoid ALND. Recent studies support this strategy: data from the Dutch MARI protocol study showed that omission of ALND in patients with ≥4 involved nodes and a post-treatment nodal pCR resulted in excellent outcomes, including a 5-year overall survival of 95% and axillary recurrence of only 2.9% (51). Similarly, a population-based NCDB analysis in cN3b patients demonstrated no difference in overall survival among those with nodal pCR treated with SLNB alone versus ALND (52). These data support TAD as a feasible and oncologically safe option in selected high-risk patients post-delivery.

Furthermore, emerging evidence indicates that omission of regional nodal irradiation in patients who convert from cN+ to ypN0 after neoadjuvant therapy does not appear to compromise recurrence-free or overall survival. Although radiotherapy has been delivered in highly selected pregnant patients with careful dosimetric planning during early gestation (48), in this case it was not required because definitive surgery was performed after delivery, allowing standard adjuvant irradiation postpartum. Finally, as treatment paradigms evolve toward risk-adapted de-escalation, multidisciplinary evaluation remains essential to balance oncologic outcomes with long-term morbidity, particularly in complex settings such as PrBC (53).

## Conclusions

This case illustrates the complexity of managing women with HER2+ PrBC, where clinical decisions must strike a delicate balance between maternal prognosis and fetal safety. Multidisciplinary care involving oncology, obstetrics, neonatology, breast surgeons, radiology, and pathology remain critical to achieve optimal outcomes. Genomic profiling tools, such as HER2DX, can guide tailoring therapy intensity. The case also highlights the diagnostic challenges of assessing tumor response in the gravid breast, emphasizing the need for careful clinical judgment and the use of appropriate imaging techniques postpartum.

The successful application of TAD, enabled by early lymph node marking, further demonstrates the importance of multidisciplinary coordination to optimize surgical outcomes, even in the context of pregnancy. Moreover, emerging evidence from small series of selected patients with excellent response to neoadjuvant treatment further support the omission of regional nodal irradiation, reinforcing the paradigm shift toward risk-based treatment de-escalation, aiming to maintain oncologic outcomes while reducing long-term morbidity.

Ultimately, this case illustrates how integrating personalized genomic data with multidisciplinary decision-making can guide management in rare and complex scenarios such as PrBC. While inherently exploratory, our experience suggests that intensified, genomically informed systemic therapy, with deferred anti-HER2 agents during pregnancy, can be delivered safely without compromising oncologic intent. Importantly, the final locoregional strategy also reflected the patient’s strong preference for minimizing axillary morbidity, and the achievement of a nodal pCR allowed consideration of a conservative approach with TAD rather than ALND. Although longer follow-up and larger studies are needed to validate this strategy, this individualized plan balanced patient priorities, treatment morbidity risk, and oncologic and fetal safety, seeking the best possible chance for durable disease control.

## Data Availability

The data supporting the conclusions of this article are not publicly available due to patient confidentiality and ethical restrictions, but are available from the corresponding author upon reasonable request.
